# Olive Polyphenols: Antioxidant and Anti-Inflammatory Properties

**DOI:** 10.3390/antiox10071044

**Published:** 2021-06-29

**Authors:** Monica Bucciantini, Manuela Leri, Pamela Nardiello, Fiorella Casamenti, Massimo Stefani

**Affiliations:** 1 Department of Experimental and Clinical Biomedical Sciences, University of Florence, Florence 50134, Italy; manuela.leri@unifi.it (M.L.); massimo.stefani@unifi.it (M.S.); 2 Department of Neuroscience, Psychology, Drug Research and Child Health, University of Florence, Florence 50134, Italy; pamela.nardiello@unifi.it (P.N.); fiorella.casamenti@unifi.it (F.C.)

**Keywords:** EVOO polyphenols, oxidative stress, inflammation

## Abstract

Oxidative stress and inflammation triggered by increased oxidative stress are the cause of many chronic diseases. The lack of anti-inflammatory drugs without side-effects has stimulated the search for new active substances. Plant-derived compounds provide new potential anti-inflammatory and antioxidant molecules. Natural products are structurally optimized by evolution to serve particular biological functions, including the regulation of endogenous defense mechanisms and interaction with other organisms. This property explains their relevance for infectious diseases and cancer. Recently, among the various natural substances, polyphenols from extra virgin olive oil (EVOO), an important element of the Mediterranean diet, have aroused growing interest. Extensive studies have shown the potent therapeutic effects of these bioactive molecules against a series of chronic diseases, such as cardiovascular diseases, diabetes, neurodegenerative disorders and cancer. This review begins from the chemical structure, abundance and bioavailability of the main EVOO polyphenols to highlight the effects and the possible molecular mechanism(s) of action of these compounds against inflammation and oxidation, in vitro and in vivo. In addition, the mechanisms of inhibition of molecular signaling pathways activated by oxidative stress by EVOO polyphenols are discussed, together with their possible roles in inflammation-mediated chronic disorders, also taking into account meta-analysis of population studies and clinical trials.

## 1. Introduction

The increasing extension in life expectancy of humans in advanced countries matches a higher prevalence of a number of lifestyle- and age-associated pathological conditions such as cancer, systemic and neurodegenerative diseases, amyloid diseases, particularly Alzheimer’s disease (AD) and Parkinson’s (PD) disease, cardiovascular diseases (CVDs) and metabolic diseases including metabolic syndrome (MetS); the latter includes, in addition to type 2 diabetes mellitus (T2DM), CVDs and non-alcoholic hepatitis. These pathologies are characterized by several common features, including, among others, derangement of proteostasis and the redox equilibrium and a remarkable inflammatory response that heavily impair the biochemical and functional features of the affected tissues. Moreover, at present, these pathologies, particularly amyloid diseases, lack effective therapies; it is then evident that, in the light of the latter aspect, prevention appears as the best tool to reduce the risk of these pathological conditions. Accordingly, medical research has progressively focused on the importance of lifestyle. Physical exercise, mental activity and diet, intended as the complex of foods and nutrients taken daily by a person, are three pillars of a healthy lifestyle.

The Mediterranean diet (MD) has been the subject of a huge amount of studies on its properties to prevent different chronic-degenerative diseases from the first evidence from the early 1960s suggesting an association between the alimentation of Mediterranean people and their low cardiovascular mortality [1]. An increasing number of epidemiological and observational studies confirm that the Mediterranean diet (MD) is associated with aging well, a condition where the prevalence of diseases including MetS, CVDs, cancer and cognitive decline appears significantly reduced [2]. The MD can be considered as the heritage of a complex socio-economic development of the Mediterranean populations over past centuries, and includes practices resulting from agricultural, social, territorial and environmental factors intimately associated with the culture and lifestyle of these populations. Recently, modifications of the classical MD have been proposed by the Mediterranean Diet Foundation Expert Group [3]. The new MD pyramid, in addition to the presence of a specific content of characteristic foods (low meat/fish, high fruit, vegetables and carbohydrates, presence of red wine, use of olive oil as main lipid source, moderate caloric intake), also emphasizes the importance of other lifestyle-associated elements, such as moderation, seasonality, adequate rest, conviviality, and physical exercise. The new pyramid also reflects the changes that the MD is undergoing, at the present, within the Mediterranean societies in relation to various geographical, cultural and socio-economic contexts. The high value of the MD and its associated lifestyle was recognized in 2010 by UNESCO, who inscribed the MD in the list of the Intangible Cultural Heritage of Humanity (https://ich.unesco.org/en/RL/mediterranean-diet-00884, accessed on 2013).

An important feature of the MD is the daily consumption of a vast array of phytonutrients including vitamins and plant phenols, which provides its similarities with the Asian diet. In particular, plant polyphenols interfere with multiple signaling pathways involved in protein homeostasis, in the inflammatory response, and in the regulation of both metabolism and the antioxidant defenses [4,5,6], often recalling a caloric restriction (CR) regimen positively affecting, among others, whole body metabolism, mitochondrial turnover, oxidative stress and the inflammatory and neuroinflammatory response, where autophagy plays an important role [7,8]. Polyphenols can reach these effects by counteracting, at the molecular level, signaling pathways responsible for the cascade reactions involved in aging [9,10]. Overall, present data support the idea that different plant polyphenols, including those from the olive tree, are able to mimic CR effects and to modulate the expression of pro- and anti-apoptotic factors, also through epigenetic modifications [11], thus affecting the same, or very similar, cellular targets. Accordingly, plant polyphenols can be proposed as a useful tool for the prevention and/or treatment of aging-associated diseases connected with chronic inflammation or transcriptional, redox or metabolic derangement [12].

An increasing number of preclinical studies, population studies and clinical trials suggest that adherence to the MD, with particular emphasis on its content of plant polyphenols, often referred to as biophenols, reduces metabolic pathologies and aging-associated deterioration, where derangement of redox homeostasis and an excessive inflammatory response often play pivotal roles. Biophenols are found in many foods of plant origin that play pivotal roles in the MD, including red wine, extra virgin olive oil (EVOO), green tea, spices, berries and aromatic herbs. The content of polyphenols in these foods and their bioavailability are quite low; however, the daily consumption, throughout one’s lifetime, of these foods ensures a reduced, yet continuous, intake of polyphenols, providing a rationale for the association between the dietary content of the latter and a significant reduction in the incidence of aging-associated pathologies reported by many population/epidemiological studies and clinical trials [13,14].

A wealth of recent studies has highlighted the fact that, in several aging-associated pathologies such as amyloid diseases, CVDs and MetS, plant polyphenols do not simply interfere with a single step of disease pathogenesis (protein/peptide aggregation, the inflammatory response, the redox/metabolic equilibrium, the proteostasis balance); rather, their positive biological and functional outcomes result from multi-target effects leading to the restoration of altered homeostatic systems in cells and tissues. In addition, the chemical similarities of these structurally distinct molecules can explain why they can induce similar effects. Among others, the importance of natural polyphenols for health has been associated with their remarkable antioxidant power elicited through the modulation of oxidative pathways. The latter can result from interference with enzymes, proteins, receptors, transcription factors and several signaling pathways [4,15]. The ability of plant polyphenols to interfere with biochemical homeostasis has also been taken into consideration [14], and epigenetic modifications of chromatin have been reported to also be involved in these effects [16,17]. Actually, recent research is providing increasing information on the biochemical, cellular and epigenetic modifications induced by several plant polyphenols and the resulting modulation of the homeostasis of key cellular processes such as metabolism, energy balance, redox equilibrium, proteostasis, cell signaling, the inflammatory response, and the control of oxidative stress and of gene expression. The knowledge stemming from these data will allow us to better understand the beneficial effects, for human wellness, of the MD, the importance of its content in plant polyphenols and the role of the latter in disease prevention and, possibly, therapy.

The rising interest in natural polyphenols has resulted in a large number of studies on their medicinal efficacy, carried out not only in cultured cells but also in model organisms and in humans. More recently, an increasing number of studies have also appeared on the biochemical and biological effects of olive polyphenols. The polyphenols elaborated by the olive tree (*Olea europaea*) are present prevalently in the leaves and drupes of the tree and are important as phytoalexins, molecules that the plant elaborates for defense against invasions by microbes and fungi and to discourage leaf-eating insects. EVOO contains over 30 phenolic compounds, including the most represented oleuropein, both in the glycated and in the aglycone (OLE) form, verbascoside, oleocanthal, hydroxytyrosol (HT), tyrosol, and others (see next section). The healthy value of EVOO and olive leaf extracts has been recognized for a long time and scientifically investigated in the last couple of centuries. More recent studies have focused on the biological properties of these molecules, including the antimicrobial, hypoglycemic, vasodilator, antihypertensive, antioxidant and anti-inflammatory ones, whose clinical importance was first reported in 1950 [18]. These properties have led to the inclusion of the alcoholic extract (80%) of olive leaves containing, in addition to minor components, OLE, HT, caffeic acid, tyrosol, apigenin and verbascoside in the European Pharmacopoeia (Ph. Eur.) [19,20].

The molecular determinants of the protection by olive polyphenols against several aging-associated and chronic degenerative conditions, including T2DM [21,22,23] and non-alcoholic fatty liver disease [24,25,26,27,28], have been extensively investigated in the last 20 years. OLE, HT and other olive polyphenols protect cells against oxidative damage resulting from redox dyshomeostasis [29,30] and an excessive inflammatory response [31], among the main determinants of age-related pathologies such as cancer, T2DM, MetS, osteoporosis and neurological diseases [27,32]. Most of these effects have been associated with the ability of polyphenols to control cell signaling and pathways, to modulate the activity of transcription factors, and to affect gene expression; these nutrigenomic properties of EVOO polyphenols have been recently reviewed [33,34]. Finally, population studies have provided evidence of a significant association between MD, EVOO consumption, and reduced risk of both CVD [35] and cognitive decline [2]. A recent review of the scientific literature focused on clinical trials and population studies has confirmed that the MD and the fortification of the foods with olive leaf extracts protect significantly against several aging-associated degenerative diseases and cancer [36,37,38]. Accordingly, plant polyphenols are increasingly taken into consideration, as such or their molecular scaffolds, as the starting component to develop new drugs especially designed to combat several chronic degenerative pathologies, including aging-associated neurodegeneration [36,39].

Here, the results of studies on the polyphenols produced by the olive tree and found in EVOO will be reviewed, with a special focus on the antioxidant and anti-inflammatory properties of these molecules. The effects of olive polyphenols in cell and animal models of aging-associated pathologies, including CVDs, MetS and neurodegenerative diseases, the molecular mechanisms underlying these properties, the currently available population studies and clinical trials, and the most recent advances in their possible use to combat neurodegenerative diseases will also be treated.

## 2. Olive Polyphenols: A Group of Molecules with Shared Chemical and Biological Properties: Structure, Abundance and Bioavailability

Biophenols are a family of over 8000 polyphenolic structures (those presently described) found in almost all plant families mainly as secondary metabolites, including several hundred isolated from edible plants [36,40]. These molecules include non-flavonoids or flavonoids; the latter are further classified as flavonols, flavononols, flavones, anthocyanins, procyanidins, phenolic acids, stilbenes and tannins on the basis of the number of hydroxyls in the molecule and the type and the position of other substituents [41]. The plant sources of plant polyphenols are, among others, bark, leaves, fruits, spices, berries, vegetables, roots, nuts and seeds, herbs, and whole grain products, from which they are transferred in processed foods of plant origin, including EVOO, red wine, green tea, coffee and turmeric. These compounds are characterized by a broad spectrum of biological activities and exert positive effects in a large number of human diseases, including cancer, CVDs, T2DM and neurodegenerative conditions, with molecular mechanisms often related to their antioxidant activity. In the case of EVOO, its healthy properties have been associated with its peculiar chemical composition [42]. EVOO contains both major components (triglycerides and other fatty acid derivatives where mainly monounsaturated fatty acids, in particular oleic acid, are present) and minor components (over 230 different chemicals including aliphatic and triterpenic alcohols, phytosterols, hydrocarbons, tocopherols, and polyphenols) [43]. In the past, the health effects of EVOO were attributed mainly to the presence of oleic acid; however, more recently, attention has been focused on phenolics, a class of bioactive compounds including phenolic acids, phenolic alcohols, flavonoids, secoiridoids and lignans [44].

In particular, olive tree polyphenols include flavonols, lignans and glycosides. Olive glycosides are iridoids, geraniol-derived monoterpenes, whose chemical structure results from a cyclopentane ring fused to a six-member heterocycle with an oxygen atom. In particular, the bicyclic H-5/H-9β, β-*cis*-fused cyclopentanepyran ring system is the most common structural feature and the basic skeletal ring of iridoids. Cleavage of the cyclopentane ring of iridoids produces seco-iridoids, while cleavage of the pyran ring produces iridoid derivatives [45]. Iridoids and secoiridoids, mainly in the glycated form, are found in many medicinal plants belonging to the subclass Asteridae that includes several plant families, particularly Oleaceae.

**Figure 1:**
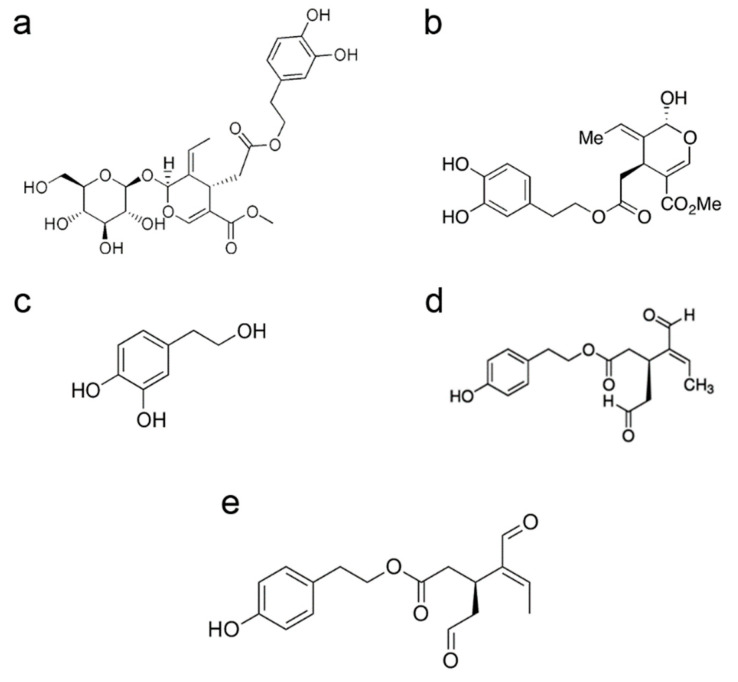
Chemical structure of oleuropein (**a**), oleuropein aglycone (**b**), hydroxytyrosol (**c**), oleacein (**d**), oleocanthal (**e**).

The polyphenols produced by the olive tree are found in the lipid fraction and in the water fraction (dispersed as minute droplets) of olive oil mainly in the glucose-free form (aglycones), resulting from deglycosylation by plant glycosidases during olive squeezing. The most abundant secoiridoid in olive oil is 3,4-dihydroxyphenylethanol-elenolic acid (3,4-DHPEA-EA), whose glucose-bound form is commonly known as oleuropein; the latter is the main cause of the bitter taste of olive leaves and drupes. Other secoiridoids include oleuropein derivatives, both in the glucose-bound form or as aglycones, such as the dialdehydic form of decarboxymethyl elenolic acid bound to either HT (3,4-dihydroxyphenylethanol-elenolic acid dialdehyde, 3,4-DHPEA-EDA, also known as oleacein) or to tyrosol (p-hydroxyphenylethanol-elenolic acid dialdehyde, p-HPEA-EDA, also known as oleocanthal, or ligstroside aglycone [46,47] (Figure 1). Oleocanthal produces the burning sensation in the back of the throat that accompanies the consumption of freshly squeezed EVOO. Olive oil also has a rich composition in simple phenols; these include tyrosol (p-hydroxyphenylethanol, p-HPEA) and hydroxytyrosol (3,4-dihydroxyphenylethanol, 3,4-DHPEA, DOPET), two phenolic alcohols mostly derived from their secoiridoid precursors. Olive polyphenols also include verbascoside, the caffeoylrhamnosylglucoside of HT, 1-acetoxypinoresinol and pinoresinol (two lignans).

Olive polyphenols are considered to be responsible for some of the recognized pharmacological properties of the olive tree (anti-atherogenic, antihepatotoxic, hypoglycemic, anti-inflammatory, antitumoral, antiviral, analgesic, purgative and immunomodulatory activities) [28,48,49], together with the protection against aging-associated neurodegeneration [29]. For these reasons, the EVOO quality depends not only on the content in free fatty acids resulting from triacylglycerol breakdown (acidity), but also on its content in polyphenols, the molecules responsible for its taste and for many of its healthy properties. Several factors affect the content of polyphenols in olive oil; these include olive cultivar, environmental cues (altitude, meteorological factors and irrigation), cultivation practices, and ripening stage of the fruits [50], together with extraction techniques, systems to separate oil from olive pastes. The conditions of storage (temperature, time) are also of importance, affecting the rate of oxidation/photooxidation reactions and the deposition of suspended water particles rich in polyphenols [51]. Under optimal conditions, the content of polyphenols in EVOO can exceed 60 mg/100 g.

The normal daily dietary intake of plant polyphenols is in the 0.1–1.0 g range; however, the bioavailability of these molecules, including the olive ones, in humans is poor due to reduced intestinal absorption and fast biotransformation that favors their urinary excretion. In addition, in the case of the brain, the circulating polyphenols must also cross the blood–brain barrier before reaching the parenchyma. With few exceptions, polyphenol aglycones can be partially absorbed in the small intestine by passive diffusion [52] much better than their glycated counterparts [53], although important amounts proceed to the large intestine to be eliminated [54]. A review of many studies on polyphenol bioavailability reported a 0 to 4.0 µmol/L plasma concentration of total metabolites produced from the oral administration of 50 mg aglycone equivalents of a polyphenol [55]. After intestinal absorption and passage to the lymph, most polyphenols undergo phase I and phase II metabolism, with substantial biotransformation and production of methylated, sulphated, hydroxylated, thiol-conjugated and glucuronide derivatives and degradation products [56]. These modifications alter the chemical properties of plant polyphenols, favor their excretion and, possibly, provide them new biological activities [57]. The importance of the colonic microflora for polyphenol bioavailability, due to its ability to metabolize and chemically modify polyphenols, has been reported recently [55]. Anyway, recent studies indicate that plant, including olive, polyphenols are absorbed in discrete amounts from the intestine and rapidly distributed through the blood flow to the whole organism, including the brain, both in rats [58,59] and in humans [60,61]. Plant polyphenols do interact with, and cross, synthetic and cell membranes. The interaction of oleuropein aglycone with synthetic phospholipid membranes favored by the presence of anionic lipids has been reported in a very recent study [62]. Another study reported that several polyphenols (the olive ones were not included) protected the mitochondria against membrane permeabilization by amyloid oligomers, suggesting some interference with oligomers’ interaction with the membrane [63]. Finally, oleuropein aglycone (OLE) was the main polyphenol found in breast cancer cells treated with an olive leaf extract in a recent metabolite-profiling study, suggesting its ability to cross the plasma membrane of these cells [64].

Due to the rising interest in natural phenols as possible new drugs, strategies to improve their bioavailability are under study, with encapsulation being probably the most actively investigated, in some cases with encouraging results [65,66]. Most of these molecular tools have not been tested in clinical trials, yet this strategy appears promising to improve the efficacy of natural phenols as drugs while reducing the amount of the administered dose. Actually, accurate studies on the effective dose of olive polyphenols to be administered daily to humans to obtain significant protection are still lacking; at any rate, the amount of OLE and other plant polyphenols taken daily in foods appears not adequate to ensure a dose suitable to produce short-term acute effects. However, clinical and experimental evidence indicates that a continuous consumption of moderate amounts of these molecules can be effective in the long term; this can also result in the accumulation in body tissues of these lipophilic molecules, leading to a low-intensity continuous stimulus of cell defenses against amyloid deposition, protein and metabolism dyshomeostasis, oxidative stress and other alterations underlying age-associated pathologies. These effects, although not proven experimentally, could, at least in part, explain the healthy properties of the MD. Nevertheless, the intake of moderate amounts of olive, and other plant, polyphenols provided by a typical MD supports the usefulness of the integration of polyphenol-enriched olive leaf extracts and other polyphenol-enriched nutraceuticals that can intensify, in the short term, the beneficial effects of these molecules.

## 3. Antioxidant and Anti-Inflammatory Properties of Olive Polyphenols in Animal Models

It is widely recognized that oxidative and nitrosative stress as well as inflammation are the major abnormalities underlying neurodegeneration and that antioxidant molecules, such as olive oil polyphenols, restore neuronal function through the amelioration of the redox status. Some beneficial effects of the MD have been associated with the consumption of EVOO polyphenols; these include antioxidant, hypoglycemic, antimicrobial, antiviral, antitumor, cardioprotective, neuroprotective, antiaging and anti-inflammatory activities [67,68]. It has been reported that EVOO polyphenols are protective against cognitive impairment associated with aging and neurodegenerative diseases due to their ability to protect DNA against oxidative stress, to inhibit mitochondrial dysfunction and to attenuate lipid peroxidation by scavenging free radicals, thus sustaining endogenous antioxidant stability [69,70]. They are also able to inhibit amyloid β (Aβ) and τ protein aggregation and toxicity, the main causes of the neurodegenerative cascade in AD [39,71,72]. EVOO polyphenols participate in the redox balance of the cell as antioxidants and as mild pro-oxidants, with ensuing upregulation of the antioxidant defenses of the cell. Accordingly, they can be considered as hormetic factors. For instance, in the presence of peroxidases, HT can undergo a redox cycling that generates superoxide [70], and tyrosol also increases *C. elegans* lifespan by activating the heat shock response [71]. It was reported that HT reduces brain mitochondrial oxidative stress and neuroinflammation in AD-prone transgenic mice by the induction of Nrf2-dependent gene expression [72]. The eight-week administration of oleuropein (60 mg/kg/day) improved mitochondrial function and reduced oxidative stress by activating the Nrf2 pathway in SHR rats [73]. Furthermore, tyrosol (240 mg/kg) was found to be protective against LPS-induced acute lung injury *through the* inhibition of NF-κB and the activation of AP-1 and of the Nrf-2 pathway [74]. EVOO polyphenols also enhance Nrf-2 activation at the hepatic level and the ensuing release of antioxidant enzymes [75]. Nrf2 is considered the principal regulator of redox homeostasis and its activation inhibits pro-inflammatory mediators such as cytokines, COX-2 and iNOS [76]. EVOO polyphenols limit inflammation by reducing the expression/activity of the transcription factors NF-κB and AP-1 [77] thanks to their free radical scavenging and radical chain breaking capacity and to the reduced formation of ROS and RNS. Moreover, HT inhibits the development of the inflammatory cascade following LPS and carrageenan injection through downregulation of the levels of pro-inflammatory cytokines (TNF-α and IL-1β), COX2, iNOS, NO, PGE2 and NF-kB and reducing DNA damage [78,79,80]. It was reported that the co-injection of OLE (450 µM) with Aβ42 (50 µM) into the nucleus basalis magnocellularis (NBM) of adult rats interfered with Aβ aggregation and significantly counteracted Aβ toxicity against choline acetyltransferase-positive neurons of the NBM and reduced astrocyte and microglia activation [81]. Another study reported that OLE protects transgenic *C. elegans* strains, constitutively expressing Aβ3-42, by reducing Aβ plaque load and motor deficits [82]. Interestingly, significant anti aggregation and neuroprotective effects of a diet supplemented with OLE, HT or a mix of polyphenols from olive mill wastewater were reported in the TgCRND8 mouse model of Aβ deposition. In these transgenic mice, a significant improvement in cognitive functions and a significant reduction in Aβ plaque number, size, and compactness were found in 3- and 6-month-old mice (at the early and intermediate stage of Aβ deposition, respectively) fed for 8 weeks with the OLE-supplemented diet [83,84,85]. A significant improvement in synaptic function and a significant reduction in the number, size and compactness of both Aβ42 and its 3-42 pyroglutamylated derivative (pE3-Aβ) deposits occurred even when the treatment was started at 10 months, when these mice display increased brain deposits of Aβ and, in particular, of pE3-Aβ in the cortex and hippocampal areas. These data indicate that oral diet supplementation with OLE not only results in the prevention of amyloid deposition but also in the disaggregation of preformed plaques and in a reduction in pE3-Aβ generation [85]. The effect of OLE against Aβ peptide aggregation was dose-dependent and could be reproduced by diet supplementation with a mix of polyphenols from olive mill wastewater or by HT administered at the same dose as that of pure OLE [84,86]. Interestingly, the treatment with OLE (50 mg/kg of diet for 8 weeks) astonishingly activated neuronal autophagy even in TgCRND8 mice at an advanced stage of pathology. In these animals, histone 3 acetylation on lysine 9 (H3K9) and histone 4 acetylation on lysine 5 (H4K5) were increased in the cortex and the hippocampus; such an increase matched both a decrease in HDAC2 expression and a significant improvement in synaptic function [85].

It is known that abnormal acetylation takes place in memory and learning disorders such as AD, where a significant increase in HDAC2 inhibits gene expression at specific loci, such as those involving autophagy markers [87]. In addition to the induction of an intense and functional autophagic response in the cortex, other relevant biological effects of OLE were uncovered in the TgCRND8 model; these include increased microglia migration to the plaques for phagocytosis, enhanced hippocampal neurogenesis and reduced astrocyte reaction [83,88]. OLE induced autophagy through the increase in cytosolic levels of Ca^2+^ and the subsequent activation of the enzyme complex AMPK by Ca^2+^/Calmodulin Protein Kinase Kinase β (CaMKKβ) and the ensuing increase in phosphorylation of mammalian target of rapamycin (mTOR) with mTOR inhibition [89]. These data support the idea that autophagy activation by OLE and other olive polyphenols proceeds via modulation of the AMPK–mTOR axis, similarly to data reported for other plant polyphenols [90]. TgCRND8 mice fed with a diet supplemented with OLE or HT (50 mg/kg of food) exhibited increased levels of Beclin-1 and LC3 autophagic markers in the soma and dendrites of neurons of the somatosensory/parietal and entorhinal/piriform cerebral cortex, together with improved autophagosome/lysosome fusion [83,86]. Furthermore, the significant accumulation of PAR polymers and the increase in PARP1 expression found in the cortex at the early (3.5 months) and intermediate (6 months) stage of Aβ deposition in the TgCRND8 mice were rescued to control values by OLE supplementation. OLE-induced reduction in PARP1 activation was paralleled by the overexpression of SIRT1, and by a decrease in the pro-inflammatory NF-κB and the pro-apoptotic p53 marker [88].

The ability of EVOO polyphenols to modulate the action of NF-kB was observed both in vitro and in vivo in different tissues. In vivo, HT attenuated apoptosis in rat brain cells by modulating the levels of caspase-3 and NF-kB p65 subunit [91]; in high-fat diet (HFD)-fed C57BL/6 J male mice, daily doses of HT (5.0 mg/kg) attenuated the increment of NF-κB and SREBP 1c, and increased the activity of Nrf2 and PPAR-γ in the liver [92]. In female BALB/c mice, an EVOO-supplemented diet was protective in the management of induced systemic lupus erythematosus disease, likely through the inhibition of the MAPK, JAK/STAT, and NF-κB pathways in splenocytes [93]. One of the most studied upstream constituents of the NF-κB signaling pathway is the activation of the mitogen-activated protein kinases (MAPKs) [94]. In the TgCRND8 mice, an HT-supplemented diet modulated MAPK signaling by activating ERK and downregulating SAPK/JNK expression, a mechanism that may underlie memory improvements in these mice [86]. These data agree with other findings suggesting an involvement of ERK stimulation in memory formation and synaptic plasticity. In the C57BL/mouse model of AD, the administration of HT and its acetylated derivative significantly improved spatial memory deficits induced by the intracerebral injection of Aβ42 plus ibotenic acid. The latter affected the Bcl-2/Bad levels, activated caspase/cytochrome-dependent apoptosis, and downregulated pro-survival genes also involved in memory functions (Sirt-1, CREB, and CTREB target genes), whereas HT administration alleviated these alterations [95]. Taken together, these data suggest that OLE and/or its metabolite, HT, can be effective to combat cellular alterations underlying AD symptoms in the absence of undesirable side effects.

Finally, HT was shown to inhibit the toxicity associated with α-synuclein aggregation in PD [96]; HT and OLE improved spatial working memory and energetic metabolism in the brain of aged mice [97]; and HT decreased oxidative stress in the brain of *db/db* mice, a widely used human T2DM animal model, by improving mitochondrial function and inducing phase II antioxidative enzymes through the activation of the Nrf2–ARE pathway [98].

To date, less data have been reported for oleocanthal. Recently, in vitro and in vivo studies reported that oleocanthal enhances β-amyloid clearance as a potential neuroprotective mechanism [99,100].

## 4. Antioxidant Properties of Olive Polyphenols: Molecular Mechanisms

The overproduction of ROS correlates with lipid, protein or DNA damage involved in the onset of degenerative diseases; accordingly, cell defenses against a rise in ROS are fundamental [101]. Antioxidants inhibit oxidation; therefore, to react to oxidative stress, organisms maintain complex systems of antioxidants, primarily glutathione (GSH). Unfortunately, only a few drugs and biological molecules, such as vitamins, have been reported to act as antioxidants, yet with possible side effects [102,103].

Nowadays, researchers are focusing their attention on the antioxidant properties of natural compounds, without relevant side effects. In particular, the importance of the antioxidant activity of lipophilic and hydrophilic phenols in EVOO has emerged [104]. This fraction is physiologically produced by plants to react against the injuries produced by various pathogens or insects [28,105]. The antioxidant activity of the major phenolic components of EVOO, OLE and HT is related to their relative bioavailability with an appreciable level of absorption, fundamental to exert their metabolic and pharmacokinetic properties [49]. In molecular terms, OLE and HT, with their catecholic structure, behave as antioxidants in different ways: (i) by scavenging the peroxyl radicals and breaking peroxidative chain reactions, generating very stable resonance structures [106]; and (ii) by acting as metal chelators, therefore, preventing the copper sulphate-induced oxidation of low-density lipoproteins [107]. The activity of OLE and HT as metal chelators could be attributed to the ability of the hydroxyl groups to behave as electron donors and to the ensuing formation of intramolecular hydrogen bonds with free radicals [32]. However, the scavenging activity of OLE and HT was also assessed in non-metal oxidation systems. Indeed, data obtained in vitro highlight the ability of polyphenols to reduce the inactivation of catalase (CAT) by hypochlorous acid (HOCl); this effect protects against atherosclerosis following LDL oxidation by HOCl through apoB-100 chlorination [108]. Moreover, HT has been reported to improve the redox status of the cell by increasing the levels of GSH [109].

Recently, the oxidative damage in age-related diseases turned out to be primarily caused by reduced levels of the transcriptional Nuclear factor erythroid 2 (NF-E2)-related factor 2 (Nrf2) [110], and it was proposed as a therapeutic target for metabolic syndromes, including obesity, due to its behavior as a mediator of general adaptive responses of the cell, including proteostasis and inflammation [111,112]. However, the pivotal role of Nrf2 is involved in the regulation of protection against oxidation [113]. Following Nrf2 activation and consequently its translocation to the nucleus, Nrf2 binds to antioxidant response elements (ARE); after binding, it acts on the transcriptional expression of several antioxidant enzymes, including superoxide dismutase (SOD), c-glutamylcysteine synthetase (c-GCS), glutathione S-transferase (GST) and NADPH quinone oxidoreductase-1 (NQO1) [114].

EVOO polyphenols have been reported to interact with Nrf2 and with Nrf2-controlled enzymes. In vivo studies showed that EVOO polyphenols increased, at the mRNA level, the expression of Nrf2 and of its targets paraoxonase-2 (PON2), c-GCS, NQO1, and GST in the heart tissue of senescence-accelerated mouse-prone 8, whose diet included 10% olive oil [115]. These effects have been ascribed to HT. Indeed, a model of metabolic alterations, the high-fat diet (HFD)-fed male mice C57BL/6J, supplemented with HT (5.0 mg/kg), displayed a reduction in oxidative stress by restoring Nrf2 and the activity of the peroxisome proliferator-activated receptor-α (PPAR-α) to normal levels [116]. The same results were obtained when the same model was supplemented with the highest dose of HT (10–50 mg/kg/day), which also resulted in an increase in GST activity in the liver and in the muscle [117]. Finally, spontaneously hypertensive rats fed with OLE (60 mg/kg/day) showed increased levels of Nrf2-dependent phase II enzymes, such as NQO-1 [77]. Anyway, in spite of these and other data, the molecular mechanisms controlled by EVOO polyphenols in terms of antioxidant activity are not still clear; in fact, the reported effects were probably determined by the tissue localization of the enzyme and by the different concentrations of phenols used. Indeed, differently from previous data, in 60-day-old Wistar male rats fed with 7.5 mg/kg/day HT, oxidative stress was increased in heart tissue, probably due to the high concentration used [118]. The latter finding is not surprising; in fact, OLE and HT exert anti-proliferative and pro-apoptotic effects on tumor cells in vitro, inducing an accumulation of hydrogen peroxide mediated by the high doses [119,120].

The activity of EVOO polyphenols on Nrf2 signaling and on the levels of several antioxidant enzymes, such as γ-glutamyl-cysteinyl-ligase (γ-GCL) and SOD, was also reported in in vitro experiments with LPS-treated macrophages [121] and cancer cells [122]. Furthermore, it is widely reported that OLE and HT act on AMPK signaling, and the latter has been considered as an attractive therapeutic target for antioxidant activity. In fact, AMPK signaling plays a fundamental role in the cell defense system against ROS by direct phosphorylation of human FoxO1 (forkhead box O1) at Thr649, with the ensuing increase in FoxO1-dependent transcription of Mn-superoxide dismutase (MN-SOD) and CAT [123].

**Figure 2:**
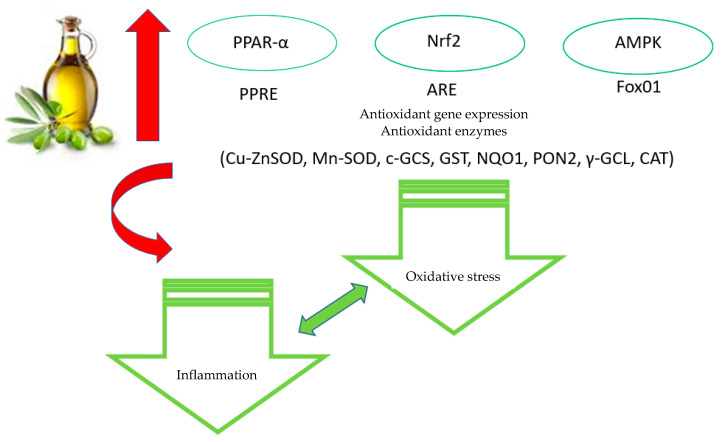
Main antioxidative effects of EVOO polyphenols.

In conclusion, the data reported in the present and in the previous paragraph convincingly support the idea that EVOO polyphenols, in particular OLE and HT, exert antioxidant activity by interfering with different cellular pathways (Figure 2).

## 5. Anti-Inflammatory Properties of Olive Polyphenols: Molecular Mechanisms

Inflammation is an essential defense mechanism of the organism by which the immune system recognizes and eliminates harmful agents and infected cells and promotes tissue repair to restore body homeostasis. This process is integrated into many coordinated functions and involves transiently elevated levels of cytokines able to activate both the innate and the adaptive immune systems. The inability to regulate an inflammatory response has multiple detrimental consequences for the organismal homeostasis; when the inflammatory response persists, a shift towards a long-term unresolved and uncontrolled immune response, known as chronic inflammation, involving macrophage- and lymphocyte-accumulated leukocytes does occur, and this results in local or systemic damage to the tissue or organs and in the degradation of normal physiologic function. Chronic inflammation is causally associated with disease onset or progression and increases with age. Indeed, the levels of cytokines, chemokines as well as the expression of genes involved in inflammation are higher in older people or in patients with autoimmune diseases that show a greater propensity to metabolic syndrome, cardiovascular disease and other chronic conditions such as frailty, multimorbidity and a decline in physical and cognitive function. Accordingly, interventions that target inflammatory pathways and restore a deregulated inflammatory response are promising strategies to prevent disease progression.

**Figure 3:**
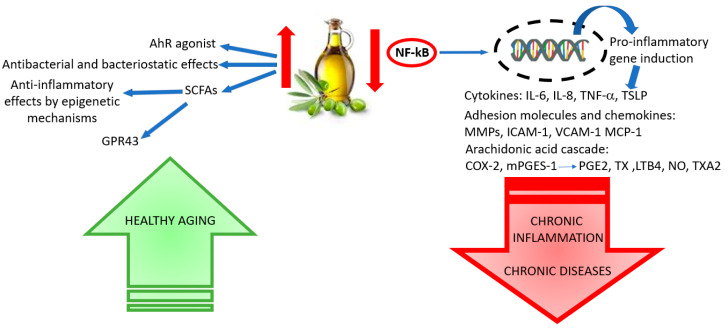
EVOO polyphenols in inflammation inhibition and their healthy effects in aging.

Convincing evidence highlights that a regular intake of food rich in polyphenols may reduce the risk for the growth of chronic diseases, including obesity, diabetes mellitus and cardiovascular diseases. This healthy effect results largely from the anti-inflammatory power of the polyphenolic compounds that is expressed by various mechanisms such as antioxidant activity (see previous paragraph) and the modulation of signaling pathways and transcriptional events (Figure 3).

In a rheumatoid arthritis model, the EVOO phenolic extracts showed joint protective properties and reduced proinflammatory mediators by the inhibition of MAPK and NF-κB signaling in activated synovial fibroblasts [124]. In this model, a polyphenolic extract also inhibited IL-1β-induced matrix metalloproteinases, TNF-α and IL-6 production, as well as IL-1β-induced cyclo-oxygenase-2 (COX-2) and microsomal PGE synthase-1 (mPGES-1) [125]. Research on the inflammatory responses in primary human keratinocytes showed that HT and its acetate ester (HTy-Ac), a natural hydroxytyrosyl derivative found in olive oil, interfere with NF-κB signaling by reducing the degradation of IκB (Inhibitor of kB), the nuclear translocation of NF-κB, its recruitment at the promoter, and the ensuing gene transcription. In addition, in this case EVOO polyphenols efficiently attenuated the expression of pro-inflammatory mediators such as thymic stromal lymphopoietin (TSLP) and the expression of several inflammation-related genes, as well as different TSLP isoforms and IL-8, thus restraining harmful processes set off by activated keratinocytes [126]. In endothelial cells, the EVOO phenolic fraction significantly reduced VEGF-induced angiogenic responses and NADPH-oxidase activity dose-dependently, resulting in the inhibition of the expression of Nox2, Nox4, MMP-2 and MMP-9 [127]. Luteolin, one of main phenolic compounds in olive oil, was able to reduce Nox4 and p22phox expression in endothelial cells treated with TNF-α and the TNF-α-induced adhesion of monocytes to human endothelial cells, a key event in the onset of vascular inflammation. The role of luteolin as an inhibitor of this inflammatory event was mediated by suppressing the expression of adhesion molecules, such as MCP-1, ICAM-1 and VCAM-1, and NF-κB signaling. Similar results were also reported with HT, tyrosol, taxifolin and OLE, which were able to inhibit angiogenesis through their inhibition of VEGFR-2 at specific phosphorylation sites [128].

In peripheral blood mononuclear cells and in endothelial cells, HT modulated the inflammatory process through a reduction in the levels of MMP-9, prostaglandin, PGE2 and tromboxanes (TX), by inhibiting COX-2 (but not COX-1). The mechanism suggested for the action of HT, tyrosol and their secoiridoid derivatives (oleacein and oleocanthal) on the inflammatory process is similar to that reported for selective inhibitors of COX-2, such as nonsteroidal anti-inflammatory drugs (NSAIDs) [110,129,130].

Recently, a protective effect, at the intestinal level, of EVOO polyphenols, in terms of the prevention of redox unbalance and of slowdown of the onset and progression of chronic intestinal inflammation, has been described in the human colon adenocarcinoma cell line (Caco-2). In these cells, the phenolic extract allowed the reversion of the oxysterols-driven activation of JNK and p38 and the following phosphorylation of IkB. The inhibition of the NF-kB pathway, iNOS induction and the reduction in IL-6, IL-8 and NO levels were detected after oxysterol stimulation in the presence of the phenolic extract. HT and its metabolites, hydroxytyrosol sulfate, 4-glucuronide and 3′-glucuronide, were able to inhibit the endothelial activation and expression of VCAM-1 and ICAM-1 in the endothelial cells of the human umbilical vein or in the intestinal Caco-2 cells stimulated by LPS or TNF-α or IL-1beta [131,132,133,134,135]. Further evidence has shown that olive oil polyphenols were particularly efficient against LPS-induced inflammation in human macrophages (THP-1 cells) by restoring a normal level of some inflammatory factors such as IL-6, IL-1β and MCP-1 [136].

Oleocanthal, in a dose-dependent manner, induced the inhibition of COX-1 and COX-2 more efficiently than ibuprofen [137]. Tyrosol and hydroxyl-isocroman compounds, a class of ortho-diphenols present in EVOO, displayed an inhibitory effect on NO release and on the arachidonate cascade and the eicosanoid synthesis (PGE2 and LTB4) in cultured macrophages (RAW 264.7) stimulated by phorbol-12-myristate-13-acetate esters [136]. 1-Phenyl-6,7-dihydroxy-isochroman, through the suppression of NF-κB activation and a decrease in COX-2 synthesis, efficiently inhibited the production of TXA2, PGE2, and TNF-α in LPS-primed human monocytes [138]. The data reported above suggest the use of HT or its derivatives as possible innovative drugs to be used in the control of inflammation and of the immune response.

The crucial role of the gut microbiota on the general inflammatory status and cardiovascular, metabolic, and even brain health is becoming more and more convincing via the gut–brain axis. Accumulating data support the beneficial efficacy of EVOO polyphenols on gut microbiota and intestinal immunity. EVOO polyphenols exhibit antibacterial and bacteriostatic effects against pathogenic intestinal microflora, improve the growth of beneficial bacterial strains, and indirectly increase the production of microbially produced short-chain fatty acids (SCFAs), which exhibit anti-inflammatory effects and modulate gene expression through epigenetic mechanisms [139,140,141]. Moreover, SCFAs are potent activators of GPR43 and play an important role in blood glucose regulation [142].

Olive oil polyphenols, such as HT and other compounds generated from certain bacterial species (e.g., Lactobacillus) favored by EVOO polyphenols, can act as ligands of the aryl hydrocarbon receptor (AhR) that represent a key element in the status of mucosal immunity and in the homeostasis of the gut barrier [143]. Furthermore, as an AhR agonist, HT was shown to favor the induction of angiogenic genes in hypoxic MCF-7 cells and to contribute to slow cancer progression and metastasis [144]. Taken together, these data suggest a significant protective effect by EVOO polyphenols at the intestinal level, supporting the link between diet and the pathogenesis and development of inflammatory bowel diseases [145]. It is worth noting that Lactobacillus and Bifidobacterium are often greatly reduced in patients with AD and in elderly people. These bacterial types, whose populations are increased following EVOO consumption, produce γ-aminobutyric acid (GABA), thus influencing the GABAergic firing pattern in the brain through enteric and vagal systems [146]. In addition, EVOO may protect cognitive performance via its antibacterial activity towards defined pathogenic species of bacteria considered as a key element for AD in the pathogen interaction hypothesis [147].

## 6. Clinical Trials Highlighting the Antioxidant and Anti-Inflammatory Properties of Olive Polyphenols

Many clinical trials and population studies provide important data on the consistent and efficacious protection resulting from a prolonged olive oil intake against the insurgence of aging-associated pathologies, such as neurodegeneration, cardiovascular diseases, metabolic diseases and cancer. Taking into consideration all the results from these epidemiological studies supporting a causal link between the intake of olive oil polyphenols and effective benefits, in November 2011 the European Food Safety Authority (EFSA) approved two health claims regarding the salutary role of olive oil consumption. The claims recommend the use of olive oil to substitute saturated fats to maintain regular blood cholesterol levels and to protect blood lipids from oxidation. These protective effects can be achieved by the intake of at least 20 g of EVOO or the consumption of 5.0 mg of HT or its derived compounds every day (e.g., oleuropein complex and tyrosol) (http://www.efsa.europa.eu/, accessed on 2012).

One of the most remarkable large dietary intervention randomized trials was the Prevención con Dieta Mediterránea (PREDIMED) trial, carried out in Spain. This trial involved 7447 participants at high cardiovascular risk, or with T2DM or ≥3 major risk factors, including smoking, hypertension, elevated LDL-C and low HDL cholesterol levels, overweight or obesity, or with a family history of premature coronary heart disease [148,149,150]. The results from this trial, at a median of 4.8 years’ follow-up, showed that the group following the Mediterranean diet supplemented with EVOO or nuts showed a 30% lower risk of developing cardiovascular pathologies, such as myocardial infarction, stroke, and consequent death, with respect to the group assigned to a low-fat diet.

In a subset of the PREDIMED trial, cognitive performance was also evaluated, with the conclusion that an EVOO-enriched MD significantly improved cognition [151,152,153]. In another subset of the PREDIMED study, the breast cancer incidence was also investigated in the same cohort. A 68% reduction in the risk of developing cancer was observed in the EVOO group [154]. In addition, results from a subsample (*n* = 990) of the PREDIMED trial indicated that a continuous intake of VOO containing a high phenolic content, instead of other types of olive oils, was efficient in preserving LDL from oxidation and in increasing the levels of HDL-cholesterol. A controlled, double blind, cross-over, randomized, clinical trial using olive oils with different phenolic concentrations (from 0 mg/L for refined olive oil, ROO, to 629 mg/L for VOO) was conducted in 30 healthy volunteers for 3 weeks, preceded by two-week washout periods. After VOO ingestion, LDL, HT monosulfate and homovanillic acid sulfate, but not tyrosol sulfate, levels were increased, while the concentrations of biomarkers of oxidative stress, including oxidized LDL (oxLDL), conjugated dienes, and hydroxy fatty acids, decreased. ROO ingestion did not affect the levels of LDL phenols and oxidation markers [155,156].

Another randomized, controlled, parallel-arm, clinical trial was carried out to compare the effects of olive oil with high (EVOO) or low (ROO) polyphenol levels in patients undergoing coronary angiography. Forty patients with at least one classic cardiovascular risk factor were randomly divided in two groups and received 25 mL EVOO or ROO daily for 6 weeks. At the end of treatment, the group that received high-polyphenol olive oil had a significant reduction in plasma LDL-cholesterol and plasma CRP. This also resulted in an increased production of inflammatory cytokines, such as IL-10, in LPS-stimulated ex vivo whole blood. Daily uptake of EVOO in subjects under pharmacological treatment could further improve LDL-cholesterol and markers of inflammation [157]. Similar beneficial effects have been demonstrated by “The Three-City Study”, carried out in 2009 on 8000 elderly subjects. This first report correlated olive oil consumption with a reduced risk of visual memory decline in a population over 65 years old [35].

The positive impact of EVOO versus low-polyphenol olive oil on markers of CVD risk in a healthy Australian cohort was investigated in a double-blind randomized cross-over study (OLIVAUS). The trial examined markers of CVD risk related to cholesterol transport and metabolism, LDL oxidation, blood pressure (peripheral and central), arterial stiffness, inflammation, and cognitive performances in 50 healthy participants subjected to three weeks of daily administration of EVOO compared to a low-polyphenol olive oil [158].

A cross-over controlled trial (ISRCTN09220811), the EUROLIVE (Effect of Olive Oil Consumption on Oxidative Damage in European Populations) study, was carried out in 25 healthy European men (20–59 years). The participants consumed 25 mL raw olive oil with low or high polyphenol content daily for 3 weeks. The interventions were preceded by a two-week washout period. Then, the effects of olive oil polyphenol intake on plasma LDL concentrations and atherogenicity were evaluated, whereas the effects on lipoprotein lipase (LPL) gene expression were checked in another subset study of EUROLIVE on 18 men. The data obtained from this study showed a decrease in plasma concentrations of apo B-100 and of total and small LDL particles together with the LDL oxidation lag time and LPL gene expression [159]. Another EUROLIVE study confirmed that olive polyphenols increase human HDL functionality, favoring HDL-mediated cholesterol efflux from macrophages [160].

The association between olive oil intake and T2DM incidence in the US population resulted from a 22-year follow-up study involving 59,930 35–65-year-old women from the Nurses’ Health Study and 85,157 26–45-year-old women from the NHS II, free of diabetes, CVDs and cancer at baseline. The results suggested that higher olive oil intake was correlated with a moderately reduced risk of T2DM, while the risk increased in women consuming other types of fats and salad dressings [161].

Another short-time study highlighted the effect of EVOO on post-prandial levels of glucose and LDL-cholesterol. Post-prandial glycemic and lipid profiles were investigated in 25 healthy subjects randomly assigned in a cross-over design to a Mediterranean diet supplemented with or without 10 g EVOO/day. The results showed that EVOO improved post-prandial glucose and LDL-cholesterol levels, suggesting an anti-atherosclerotic effect of the MD [162]. Furthermore, the same trial revealed that EVOO consumption resulted in high GLP-1 and gastric inhibitory peptide (GIP) levels in the circulatory system, while in another trial with type 1 diabetes (T1D) patients, an increase in gastric emptying and GPL-1 secretion was observed together with reduced glucose absorption through glucose–lipid competition that can contribute to a lower glycemic response [163]. In addition, an acute intake of EVOO resulted in a significant reduction in the levels of plasma glucose, triglyceride, apolipoprotein B-48, and dipeptidyl peptidase-4 activity and in a significant increase in the peripheral blood levels of insulin and glucagon-like peptide 1 (GLP-1), as revealed by a randomized trial of 30 participants with impaired fasting glucose levels [164].

The MICOIL pilot study was published on 10 November 2020. The trial confirmed that the long-standing benefits against cognitive impairment of polyphenol-enriched olive oil are greater than those granted by “simple” EVOO. The clinical trial divided participants with mild cognitive impairment (MCI) into three randomized groups. Genetic predisposition to AD was taken into account to obtain a homogenous baseline. Each group followed a unique diet: The first group received 50 mL/day of high-polyphenol olive oil, while following an MD. The second group received 50 mL/day of olive oil with moderate phenolic content, along with an MD. The third group only followed a normal MD. Long-term consumption of early harvest high phenolic or moderate phenolic EVOO was associated with an important amelioration of cognitive performance, as opposed to the low phenolic content MD, independent of the presence of genetic predisposition [165]. In 2010, a study on 20 patients with MetS showed that the acute intake of VOO was able to reduce the postprandial inflammatory response and the expression of several pro-inflammatory genes, mainly by decreasing the activation of NF-kB, of the activator protein-1 transcription factor complex AP-1, cytokines, mitogen-activated protein kinases (MAPKs) or arachidonic acid pathways, secondary to the reduction in LPS intestinal absorption following a high-fat meal [166,167].

To describe the exact role of olive oil in the metabolic changes reported above, a network meta-analysis of 30 human intervention studies totalizing 7688 subjects has been performed [168]. Using this approach, it was shown that the effect of olive oil on glycemia and blood lipids cannot be distinguished from the impact of MD adherence. Indeed, the administration of olive polyphenols in the dose suggested by EFSA does not modify glycemia levels, while it ameliorates insulin sensitivity [169]. These data are in accordance with the reported evidence of a direct action of polyphenols on the pancreas [170] and with the improvement of insulin secretion through the anti-inflammatory activity of oleic acid [171]. The only clear effect of the intake of a high-polyphenol olive oil was on HDL-cholesterol levels, on LDL and nucleic acid oxidation and on the plasma antioxidant activity, in agreement with previous meta-analyses [172].

Finally, an MD supplemented with polyphenol-rich EVOO has probiotic effects promoting the growth of bacteria of the Lactobacillus and Bifidobacterium types. These data result from different studies where overweight/obese participants and patients with HIV or with hypercholesterolemia consumed 40–50 g/day of EVOO for 12 weeks [172,173,174].

## 7. Conclusions and Future Perspectives

Several data highlight the ability of olive oil polyphenols to counteract aging and to protect against the insurgence of aging-associated pathologies, such as neurodegeneration, cardiovascular and metabolic diseases, and cancer, in part associated with derangement of redox homeostasis and proteostasis. However, recent research supports the idea that the health-promoting properties of olive oil polyphenols go well beyond their anti-amyloid and antioxidant power reported previously, highlighting their multi-target effects.

The claimed benefits of olive oil polyphenols have been supported by positive and encouraging results from many preclinical studies both in vitro and in animal models, as well as by population surveys and clinical trials often involving large numbers of participants. However, to date, there are still some doubts to resolve and therefore definitive results are lacking, even for the bioavailability of these molecules and their effective beneficial dose. In particular, further research is needed to better describe at the molecular and genetic levels the effects of olive polyphenols in several investigated biological systems to provide solid and definitive proof of their positive effects in a number of human pathologies. It must also be considered that the health benefits in humans most likely do not depend on the consumption of a single polyphenol but are the result of a variety of synergistic mechanisms of a combination of several polyphenols or other plant components.

Each factor affecting the bioavailability, bioaccessibility and bioactivity of polyphenols should also be considered. This is crucial because the bioavailability of these molecules is influenced by many factors, including phenolic structure, food processing, the food matrix, and the organism (microbiota composition, efficiency of detoxification mechanisms); furthermore, all these factors can interact with each other, modulating polyphenol bioavailability. Moreover, the latter, and thus the efficacy of these compounds, can be improved by administration in combination with other phytochemicals or drugs or in polyphenol-loaded nanotechnology-based delivery systems.

Finally, it might be more relevant and interesting to investigate the relationship between EVOO polyphenols and the gut microbiota to obtain further dietary indications. In fact, the dietary polyphenols/gut microbiota relation is a bi-directional one. On the one hand, polyphenols can affect the composition of gut microbiota; on the other hand, the microbiota is able to metabolize these molecules into bioactive compounds. Expanding knowledge on the effects of dietary polyphenols on the intestinal microbiota and the relative mechanisms of action and ensuing consequences, in addition to pharmacokinetics and pharmacodynamics of EVOO polyphenols, will be essential to better assess the effective doses and the levels reached by these molecules in different tissues and organs following different routes of introduction.
